# A Comprehensive Meta-analysis on Intra Ocular Pressure and Central Corneal Thickness in Healthy Children

**Published:** 2017-06

**Authors:** Majid FARVARDIN, Fatemeh HEIDARY, Kourosh SAYEHMIRI, Reza GHAREBAGHI, Mahmoud JABBARVAND BEHROOZ

**Affiliations:** 1. Poostchi Eye Research Center, Dept. of Ophthalmology, Shiraz University of Medical Sciences, Shiraz, Iran; 2. Eye Research Center, Farabi Eye Hospital, Tehran University of Medical Sciences, Tehran, Iran; 3. Dept. of Biostatistics and Epidemiology, Ilam University of Medical Sciences, Ilam, Iran

**Keywords:** Central corneal thickness, Intraocular pressure, Children, Correlation, Meta-analysis

## Abstract

**Background:**

Glaucoma is the major ophthalmic public health issue and a leading basis of blindness. Elevated intraocular pressure (IOP) is still a foremost risk factor in development and progression of glaucoma. Central corneal thickness (CCT) may play as the risk factor for the progression of glaucoma, closely associated with IOP especially in pediatric age group. This study performed a pioneering investigation combining the outcomes of multiple studies using a meta-analytic approach.

**Methods:**

Nineteen published articles between 1980 and 2015 were designated by searching Scopus, PubMed, and Google Scholar and analyzed with random effects model while I^2^ statistics employed to find out heterogeneity. Subsequently, the information statistically analyzed by Stata software ver. 11.20.

**Results:**

The mean IOP has been documented to 16.22 mmHg (95% CI: 15.48–16.97) in all races subgroups. Analyzing the data by race-based subgroups revealed the lowest IOP of 12.02 mmHg (95% CI: 11.40–12.64) in Indian children while IOP of 17.38 mmHg (95% CI: 15.77–18.98) documented in black children as the highest measurement. The mean CCT was 553.69 micrometer (95% CI: 551.60–555.78) among all races. Lowest CCT of 536.60 mm (95% CI: 531.82–541.38) has been documented in mixed Malay-Indian children whereas Chinese children ought to the highest CCT value of 557.68 mm (95% CI: 553.10–562.25).

**Conclusion:**

Findings of published studies were inconsistent when considered independently; however, meta-analysis of these results showed a significant correlation between CCT and IOP. Owing to non-uniform methods used to measure IOP and CCT in studies, data were stratified into various subgroups according to the instruments used to measure IOP and CCT.

## Introduction

Glaucoma is a major ophthalmic public health issue that affects hundreds of millions of patients may consider as one of the prominent causes of blindness (1). Intraocular pressure (IOP) is regularly calculated and documented to monitor the progress of glaucoma while positive linear correlation between central corneal thickness (CCT) and IOP has been described in the literature (2).

Additionally, CCT is a significant value for understanding morphology of the cornea as well as for the development of various ophthalmic diseases including glaucoma. Numerous researches in children and adults revealed that IOP might be affected by the CCT measurement. Normally, a thin cornea underestimates whereas a thick cornea overestimates the IOP (3). CCT is a significant factor in the glaucoma diagnosis and treatment since having low CCT value may indicate to under-diagnosis and under-treatment of glaucoma, while a high CCT may cause to over-diagnosis and overtreatment of diseases (3). The results of some studies have indicated a relationship between IOP and ethnicity. Moreover, CCT might differ among subjects from different ethnic groups (3).

The main purpose of the current study was to reveal a meta-analysis to shed light on the relationship between CCT and IOP in children from different ethnic subgroups. To the best of our knowledge such, a meta-analysis has not been formerly performed in this field.

## Methods

Databases including PubMed, PubMed Central, SCOPUS, and Google Scholar searched for published studies related to CCT and IOP in children. The search strategy has been limited to English language publications prior to Nov 2015.

Subsequently, the publication bias test performed independently. Two authors individualistically assessed the titles of all publications, eliminating duplicate papers and classifying theoretically applicable researches to be included in analysis. Two authors for additional relevancy appraised abstracts from designated studies whereas full-text publications recovered. In the case of dissimilarity, a third appraiser corresponded to as an authority. Just in case, if the full text of a publication was not found, endeavors were made to contact directly to corresponding author by Email. Nevertheless, if this was ineffective the publication was ignored.

The following information obtained from included researches: first author, year of study, age distribution, CCT, IOP, ethnicity, relationship between CCT and IOP, and instruments used to measure CCT and IOP. The principal outcome measures of interest for this manuscript were the mean CCT and IOP, as well as 95% confidence interval and relationship between CCT and IOP.

By Mantel-Haenszel, random effect modeling data was analyzed and presented in a Forest plot. The standard error of the mean for each paper was designed using the normal distribution. For pooled correlation coefficients, the effect size defined. Following this transformation, by using random effects model effect size pooled. Heterogeneity determined by the chi-square test with a *P*-value less than 0.1 at significant level combined with an I^2^ statistic for approximations of inconsistency within the analyses. The I^2^ statistic estimated the percent of observed between study variability because of heterogeneity rather than because of chance and ranged from 0 which defined as no heterogeneity to 100% as described to noteworthy heterogeneity. Statistically, I^2^ values exceeding 75% were revealing of significant heterogeneity warranting investigation with a random effect model as opposed to the fixed effect model to adjust for the observed variability. Heterogeneity was explored through subgroup meta-regression. Univariate and multivariate approaches employed to consider the reasons for heterogeneity among the selected included publications, and subsequently the Egger test performed to inspect bias. Statistical analyses performed using Stata software ver. 11.20.

## Results

Our searching yielded 53 articles. Following exclusion of duplicates, 19 publications selected for final analysis. Totally, 47266 individuals aged less than 17 yr old participated. The descriptions of included studies are presented in Table 1 and 2.

**Table 1:** Study characteristics of intra ocular pressure (IOP) in children

**Author**	**Year**	**Country**	**Race**	**Number**	**Measurement of IOP**	**Mean IOP (mmhg)**
Heidary F^4^	2010	Malaysia	Malay	54	Air_puff noncontact tonometer	15.65
Haider MK^5^	2007	USA	Black	60	Tono_pen	16
	2007	USA	White	76	Tono_pen	15
Muir KW^6^	1997	USA	Black	27	Goldmann applanation tonometer (GAT)_Tono-Pen	19.3
			White	29	Goldmann applanation tonometer (GAT)_Tono_Pen	17.7
Muir KW^7^	2004	USA	Black	35	Goldmann applanation tonometer(GAT)_Tono_Pen	19.3
			White	52	Goldmann applanation tonometer(GAT)_Tono_Pen	17.7
Doughty MJ^8^	2001	New Zealand	White	104	Non-contact tonometer(Handheld air_puff)	16.7
Hikoya A^9^	2005	Japan	Japanese	169	Tono_Pen	13.9
Lim L^10^	2007	Singapore	Chinese	186	Non-contact tonometer(ORA)	
			Malay	50	Non-contact tonometer(ORA)	
			Indian	33	Non-contact tonometer(ORA)	
Tong L^11^	1999	Singapore	Chinese	485	Air_puff noncontact tonometer	
			Malay & Indian	167	Air_puff noncontact tonometer	
Sahin A^12^	2007	Turkey	White	165	Tono_Pen	17.47
			White	165	Rebound_Tonometer	16.81
Krzyza. B.^13^	2012	Poland	White	75	Non-contact tonometer NCT) (Air_puff)	15.9
			White	75	Icare tonometer(Rebound_Tonometer)	16.9
			White	75	Goldmann applanation tonometer(GAT)	14.7
Song Y.^14^	2002	China	Chinese	1153	Non-contact tonometer (ORA)	17
Sakalar YB^15^	2008	Turkey	White	15160	Air_puff noncontact tonometer	14.15
Huang Y^16^	2013	China	Chinese	571	Non-contact tonometer (ORA)	17.36
Bueno-G I.^17^	2014	Spain	White	99	Non-contact tonometer (ORA)-iopg	16.75
			White	99	Non-contact tonometer (ORA)-iopcc	14.71
Yildirim N.^18^	2006	Turkey	White	602	Tono_Pen	17.9
			White	602	Air_puff noncontact tonometer	16.75
PEDIG.^19^	2011	USA	White	807	Tono_Pen	
			Black	474	Tono_Pen	
			Hispanic	494	Tono_Pen	
Ramanjit S.^20^	2004	India	Indian	405	Perkins applanation tonometer	12.02
Wei W.^21^	2013	China	Chinese	514	Air_puff noncontact tonometer	15.31
Huang Y^22^	2013	China	Chinese	571	Goldmann applanation tonometer(GAT)	17.36

**Table 2:** Study characteristics of central corneal thickness (CCT) in children

**Author**	**Year**	**Country**	**Race**	**Number**	**Measurement of CCT**	**Mean CCT (micrometer)**
Heidary F^4^	2010	Malaysia	Malay	54	Specular Microscope	530.87
Haider MK^5^	2007	USA	Black	60	Ultrasonic pachymeter	535
	2007	USA	White	76	Ultrasonic pachymeter	559
Muir KW^6^	1997	USA	Black	27	Ultrasonic pachymeter	537
			White	29	Ultrasonic pachymeter	564
Muir KW^7^	2004	USA	Black	35	Ultrasonic pachymeter	543
			White	52	Ultrasonic pachymeter	562
Doughty MJ^8^	2001	New Zealand	White	104	Ultrasonic pachymeter & Specular Microscope	529
Hikoya A^9^	2005	Japan	Japanese	169	Ultrasound pachymeter	544.3
Lim L^10^	2007	Singapore	Chinese	186	Ultrasonic pachymeter	584.1
			Malay	50	Ultrasonic pachymeter	573.4
			Indian	33	Ultrasonic pachymeter	557.5
Tong L^11^	1999	Singapore	Chinese	485	Automated, noncontact optical low-coherence reflectomery(OLCR) pachymeter	546
			Malay & Indian	167	Automated, noncontact optical low-coherence reflectomery(OLCR) pachymeter	536.6
Sahin A^12^	2007	Turkey	White	165	Ultrasonic pachymeter	561.37
			White	165	Ultrasonic pachymeter	561.37
Krzyza. B.^13^	2012	Poland	White	75	Ultrasonic pachymeter	563
			White	75	Ultrasonic pachymeter	563
			White	75	Ultrasonic pachymeter	563
Song Y.^14^	2002	China	Chinese	1153	Ultrasonic pachymeter	553
Sakalar YB^15^	2008	Turkey	White	15160	Ultrasonic pachymeter	557.91
Huang Y^16^	2013	China	Chinese	571	Ultrasonic pachymeter	556.01
Bueno-G I.^17^	2014	Spain	White	99	Anterior segment OCT	543.85
			White	99	Anterior segment OCT	543.85
Yildirim N.^18^	2006	Turkey	White	602	Ultrasonic pachymeter	564.92
			White	602	Ultrasonic pachymeter	564.92
PEDIG.^19^	2011	USA	White	807	Ultrasonic pachymeter	573
			Black	474	Ultrasonic pachymeter	551
			Hispanic	494	Ultrasonic pachymeter	573
Ramanjit S.^20^	2004	India	Indian	405	Ultrasonic pachymeter	541
Wei W.^21^	2013	China	Chinese	514	Non-Contact Tono / Pachymeter	554.19
Huang Y^22^	2013	China	Chinese	571	Ultrasonic pachymeter	556.01

**Fig. 1:**
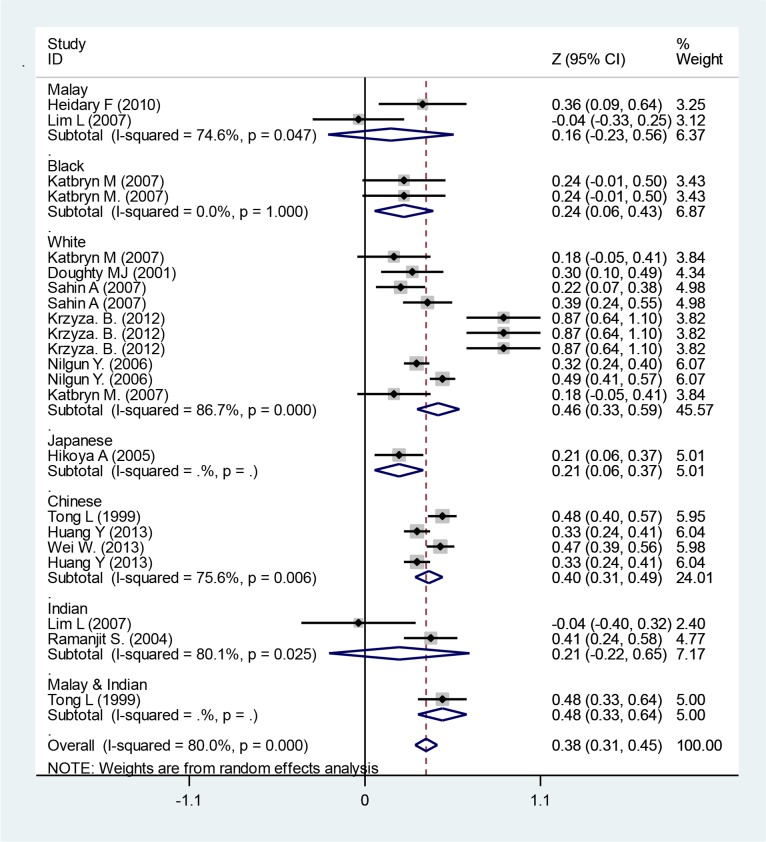
Logarithm transformation of correlation coefficients between IOP and CCT. Squares corresponded to effect estimate of outcomes with 95% confidence intervals as the size of the squares proportional to the weight allocated to the included publications. Diamonds reveal the overall outcomes and 95% confidence interval of the random effect. Lines reveal the confidence interval. Publications that do not cross the zero line show a meaningful correlation between CCT and IOP. The outcomes show a significant correlation between CCT and IOP (r=0.0, *P*=00)

**Fig. 2:**
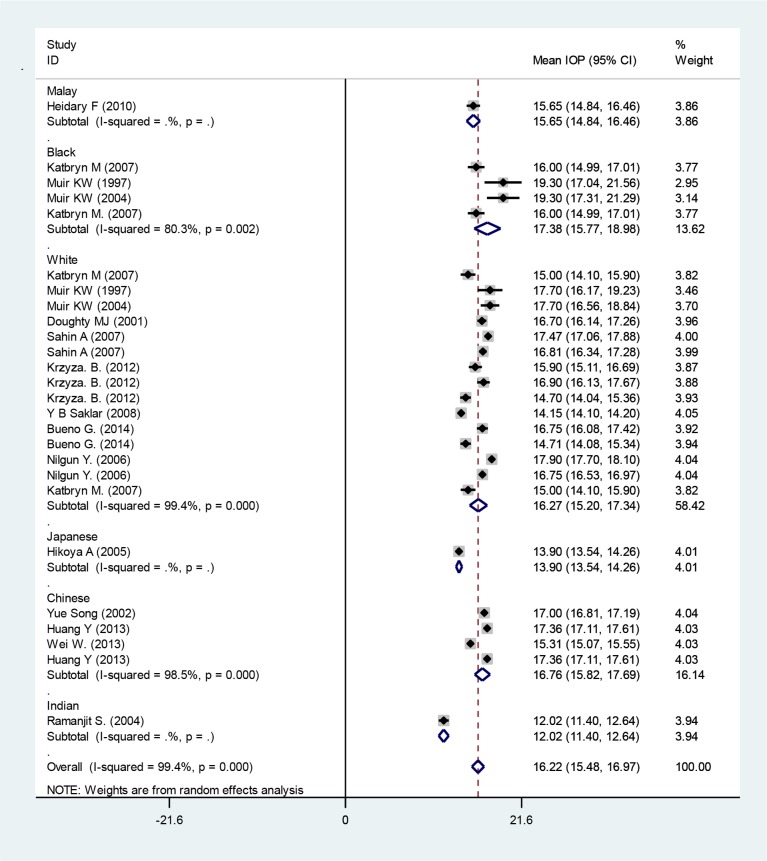
Mean IOP based on ethnicity subgroup. Squares corresponded to effect estimate of outcomes with 95% confidence intervals with the size of the squares proportional to the weight allocated to the included publications. Diamonds reveal the overall outcomes and 95% confidence interval of the random effect.

The outcomes demonstrated a significant correlation between CCT and IOP (r=0.0, *P*=00) (Fig. 1). With transformation of z to r that we were able to compute, r, 95% CI for r is 0.36 (0.30–0.43). This indicates a meaningful relationship between IOP and CCT. The mean IOP from included studies was 16.22 mmHg (95% CI: 15.48–16.97) in all races (Fig. 2). Race-based subgroups analysis revealed that Indian children with the lowest IOP of 12.02 mmHg (95% CI: 11.40–12.64), whereas black children with the highest IOP level of 17.38 mmHg (95% CI: 15.77–18.98).

**Fig. 3:**
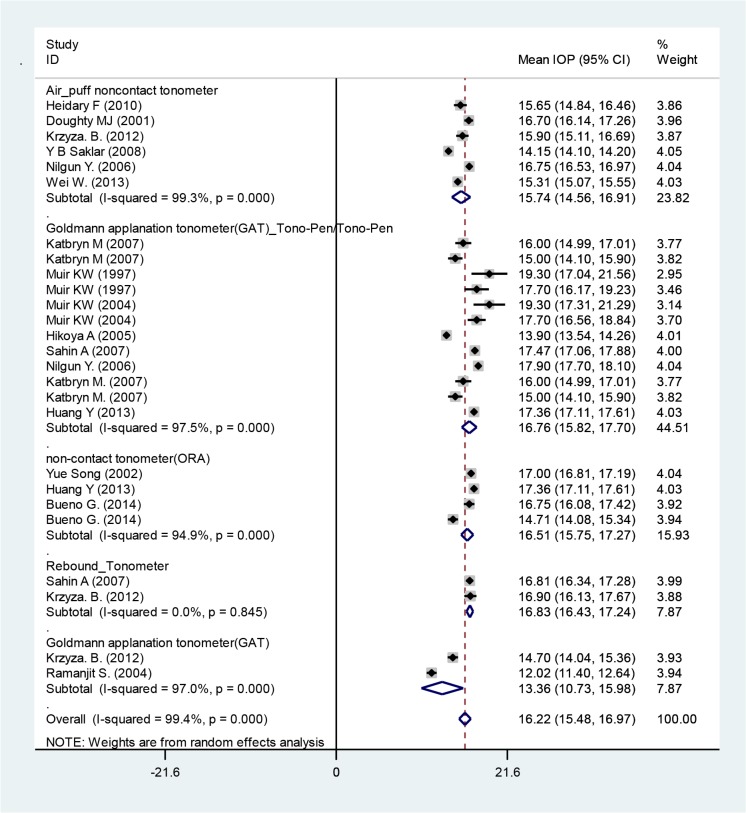
Mean IOP based on the instrument that used. Squares corresponded to effect estimate of outcomes with 95% confidence intervals with the size of the squares proportional to the weight allocated to the included publications. Diamonds reveal the overall outcomes and 95% confidence interval of the random effect.

The mean IOP from included studies was 16.22 mmHg (95% CI: 15.48–16.97) in all races (Fig. 2). Instrument-based subgroups analysis for measurement of IOP, revealed that Rebound tonometer had highest IOP measurements with mean IOP of 16.83 mmHg and Goldmann applanation tonometer(GAT) had lowest IOP measurements with mean IOP of 13.36 mmHg (Fig. 3).

**Fig. 4:**
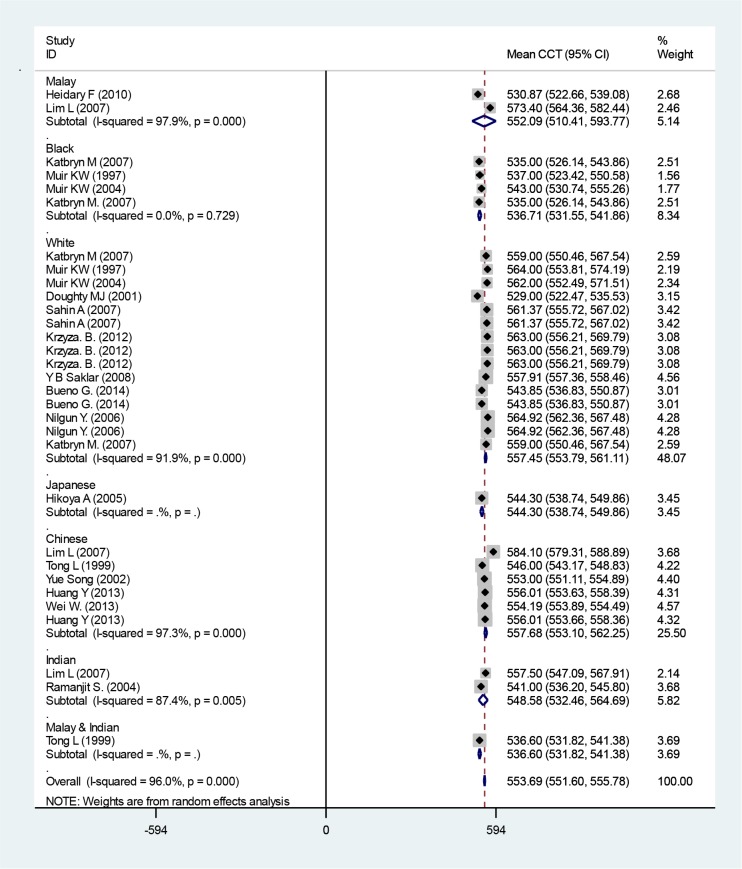
Mean CCT based on ethnicity subgroups. Squares corresponded to effect estimate of outcomes with 95% confidence intervals with the size of the squares proportional to the weight allocated to the included publications. Diamonds reveal the overall outcomes and 95% confidence interval of the random effect.

The mean CCT from all articles was 553.69 micrometer (95% CI: 551.60–555.78) (Fig. 4). Race-based subgroup analysis revealed that mixed Malay-Indian children revealed the lowest CCT of 536.60 mm (95% CI: 531.82–541.38), whereas Chinese children had the highest CCT of 557.68 mm (95% CI: 553.10–562.25).

**Fig. 5:**
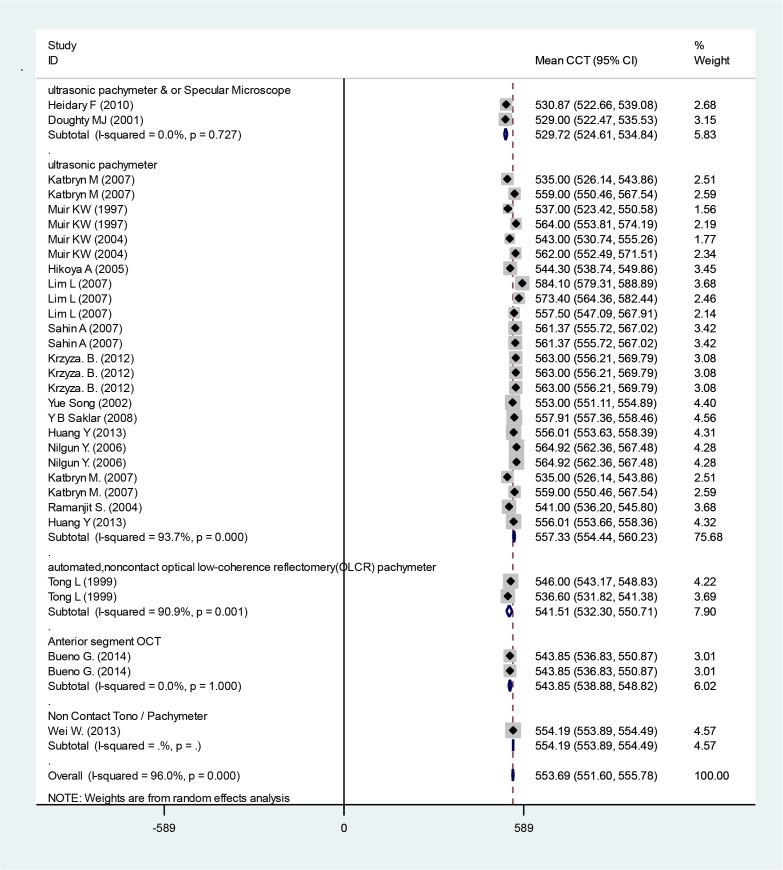
Mean CCT based on instrument that used. Squares corresponded to effect estimate of outcomes with 95% confidence intervals with the size of the squares proportional to the weight allocated to the included publications. Diamonds reveal the overall outcomes and 95% confidence interval of the random effect.

We presented the subgroups based on instruments used for measurement of CCT and IOP in Fig. 3 and 5.

The statistical evaluation for publication bias comprising Begg and Egger tests did not meaningful approving absence of publication bias in our manuscript (*P*=0.05).

## Discussion

Our results revealed that the mean IOP and CCT documented to 16.22 mmHg and 553.69 mm, respectively. The final analysis disclosed ethnicity-based differences in IOP and CCT measurement. Analyzing race-based subgroups showed Indian children with lowest IOP of 12.02 mmHg whereas black children with the highest IOP of 17.38 mmHg. Mixed Malay-Indian children presented with the lowest CCT of 536.60 mm whereas Chinese children with the highest CCT of 557.68 mm. Our research is the meta-analysis approach of CCT and IOP in children; however, since CCT and IOP measurements performed with different instruments, we were unable to compare outcomes across studies.

Such differences in mean CCT and IOP among sub-groups may offer the hypothesis of the presence of morphological and anatomical disparities among ethnicities. Goldmann applanation tonometers are thought the gold standard for measurement of IOP (5), as well as ultrasound pachymeters, reflected the gold standards in measurement of CCT. However, since children are usually uncooperative, most studies used mixed contact and non-contact methods; therefore, we were unable to compare results homogenously.

Former studies showed influence of socioeconomic status on CCT and IOP (4). The socioeconomic backgrounds or effects of environmental factors, as well as levels of malnutrition, were not documented in extracted studies, therefore, we were unable to analyze. This may merit further investigation in future studies as well as longitudinal approach in order to categorize subjects based on their level of socioeconomic status and may measure effect of environmental factors on biophysics of ocular structure.

Different instruments may yield different documentation in measurement of CCT in the same case, for instance, a measurement by specular microscopy may result meaningfully lower values than ultrasound pachymeter measurement (23). In another study, CCT measurements of different instruments were compared while finding out contact specular microscopy was substantially documented lower than measured using other instruments (24).

There is controversial issue in relationship between age and CCT. CCT gradually increases by 5 yr of age, upon which it may reach steady prior beginning to decrease at 10–14 yr of old (6). Relationship between CCT and IOP among children less than 10 yr of age was struggled, did not realize any difference in CCT among the different age subgroups (4). In our meta-analysis, most of included publications did not classify their participants into subgroups; therefore, we were unable to formulate age-based comparisons. A modification factor of 2.5 mmHg was recommended for each 50-micrometer difference in CCT (25). Actually, evidence regarding the link between CCT and IOP are controversial. Although a few studies observed no meaningful relationship between mean IOP and CCT among either African American (R=0.24) or White (R=0.18) children (5) others demonstrated the positive relationship like our analysis revealed a very significant relationship between IOP and CCT (*P*=0.00), as conclusion.

The limitation of the current study was largely associated with the methodology approach of the reviewed publications, individually. Lack of a uniform method of the measurements were the primary limitation; however, such a meta-analysis has not been formerly performed in this field considered as the strength of this research in order to summarize the findings of all related studies and reach the final conclusion regarding the mean CCT and IOP and their relationship.

Discovering of racial differences in normal ocular structures may establish invaluable reference value and may promote further understanding of various ocular disorders(26), therefore, future meta-analysis on normal ocular structure are also required.

## Conclusion

Findings of published studies were inconsistent when considered independently; however, meta-analysis of these results showed a significant correlation between CCT and IOP. Owing to non-uniform methods used to measure IOP and CCT in studies, data were stratified into various subgroups according to the instruments used to measure IOP and CCT.

## Ethical considerations

Ethical issues (Including plagiarism, informed consent, misconduct, data fabrication and/or falsification, double publication and/or submission, redundancy, etc.) have been completely observed by the authors.
